# Harmonizing conscience claims and patient access to assisted death

**DOI:** 10.1177/08404704231158701

**Published:** 2023-03-16

**Authors:** Eric Wasylenko

**Affiliations:** 1 University of Calgary, Calgary, Alberta, Canada.; 2 3158 University of Alberta, Edmonton, Alberta, Canada.

## Abstract

The rights of patients to receive legally permissible interventions sometimes conflict with enshrined rights of providers to object, for reasons of conscience, to providing those interventions. Getting the balance right is challenging. But reasonable balance to manage these conflicting imperatives can be achieved in the design of programs for assisted death. Rather than limiting the discourse to the platform of competing individual rights, health leaders are urged to consider the broader societal benefits and impacts of valuing conscience in the practice of medicine, the creation of regulation and policy, and the delivery of healthcare. A method to determine that conscience claims are “genuine,” “reasonable,” and “acceptable” needs developing. A list of criteria toward this determination is offered.

## Introduction

In compliance with the Supreme Court of Canada’s ruling in *Carter* vs. *Canada (Attorney General)*,^1,2^ legislators, regulators, and health leaders have subsequently attempted to legislate and implement a regime for assisted death that satisfies at least these three overarching imperatives:

1. Make assisted death accessible to eligible Canadians who seek it.
2. Establish robust safeguards, especially to protect vulnerable persons.
3. Harmonize clinicians’ conscience rights with the rights of citizens to access assisted death.

Addressing the third imperative, I wish to assert that the appropriate harmonization should not be discerned only by weighing the competing rights of individuals. Rather, health leaders should bear in mind additional considerations that the exercise of conscience provides in the delivery of healthcare and toward society’s benefit.

Conscience is understood as moral self-awareness. The word’s origin is from the Latin *conscientia* – *scienta* (knowledge) and *con* (with).^3^ The Greek concept of *synderesi*s refers to the moral awareness that is within a person guiding toward good acts and away from evil acts.^4^

Conscience has been described in philosophical writing and discourse from as far back as Greek and Roman playwrights in the 5^th^ century BCE, through the time of the Stoics, and throughout the subsequent 2,600 years of secular philosophy.^3,4^ It also appears in the tenets of every major religion and the writing of religious philosophers.^5,6,7,8,9^ For an in-depth exploration of the roots of conscience in secular and religious philosophical thought, I refer readers to the *Stanford Encyclopedia of Philosophy*.^3^

Conscience is formed and nourished through attention to such things as social mores, culture, teaching, experience, laws and regulations, religion, and professional codes. Individuals attribute differential weight (and sometimes no weight at all from particular of these sources) to these inputs and look to any combination of the multiple sources for guidance regarding a virtuous course of action. Conscience is an inward looking and reflective resource for an individual. Since conscience is core to an individual’s personhood, its protection serves to maintain inner coherence, or integrity of oneself.^10,11,12^ Allowing adherence to the moral convictions that comprise one’s conscience demonstrates respect for the person and their core identity. It also secures connection with the moral pillars of one’s professional identity. As a result, freedom of conscience has been enshrined in declarations of Human Rights, in the *Canadian Charter of Rights and Freedoms*, and in professions’ Codes of Ethics.^3,11,13‐17^

The freedom to form conscience about assisted death and a clinician’s participation in it is not in dispute. Of more interest is the freedom to *act in accordance with* one’s conscience while simultaneously discharging one’s professional duties in the clinical milieu.^12,17‐21,^^(note a),22^ This is the crux of the issue.

## Discussion

Some authors are committed to the idea that conscience protection is legitimate and required, and that the ability to honour one’s core beliefs is necessary to support their essential integrity.^3,4,12^ Support for our clinicians’ and leaders’ moral integrity, which forms the core of their personhood, helps them remain whole and healthy while they are occupied in their professional roles. Yet a core of the ethos of healthcare is that patients should have reasonable and equitable access to legal health services. Therefore, despite the importance of conscience adherence, there are limits in its application. For instance, a person not participating in a medical act for reasons of conscience, or one who objects to the service on moral grounds, ought not impede access to the legal service for any patient.^12,15,22,23^ The power imbalances that are inherent in healthcare compel us to assure that patients are not subjected to heightened imbalances that the exercise of conscience might entail.^11,24^

A competing view about the legitimacy of conscience protections holds that no room exists for conscientious objection regarding services such as assisted death, when a clinician acts as an agent of the state in a publicly funded health system.^18^ Rather, it is argued such a clinician must be prepared to offer, provide, or facilitate in some manner, the legal and clinically acceptable service if a patient freely chooses it, in respect of the patient’s autonomy. This also serves the modern health system’s core object of providing person-centred care. And so, legal and ethics tensions remain.

If we contemplate this issue as only a competing or co-existing “rights” issue about individuals, we are missing an additional and essential aspect of the value of paying attention to conscience, that is, the work that conscience performs in the human endeavour of healthcare.^12^

Why should we want clinicians to be able to adhere to their moral commitments? I highlight five societal benefits of valuing conscience in practice:

1. Healthcare is often challenging. While some interactions and decisions can be quite straightforward, many require deep reflection, discernment, and application of wisdom. A healthy and exercised conscience is one central resource that clinicians and leaders turn to as they guide patients and colleagues and as they provide care or organize care delivery in an exemplary manner. In the very human and moral endeavor of healthcare, a vulnerable person places their trust in the skills, competence, knowledge, and wisdom of another. Especially when the decisions to be made are challenging, and may involve life and death, we should want clinicians and leaders to act with integrity—by being authentic, establishing moral commitments, deliberating on those commitments throughout their careers, and acting in accordance with them.^12,25^
2. Some citizens may have moral objections to certain interventions and might feel most safe being cared for by clinicians who hold moral views similar to their own and who can act in harmony with their conscience.^26^
3. Conscience plays a role in discernment about current and emerging healthcare practices, including those containing moral uncertainty,^12^ sometimes serving as an early warning system that something might be amiss, even if legal.^27^ The history of medicine is replete with examples in which legal and institutionally promoted interventions or research was successfully called into question by individuals exercising their consciences. Well-known examples include eugenics laws, forced sterilization practices, genital mutilation, and medical complicity in prisoner torture. Conscience considerations regarding research into cloning and retrenchment on pharmaceutical company inducements to clinicians provide other examples. There are many more.
4. No other humans in our society are entrusted with permissions and obligations to beneficially probe another person’s mind, history, and body nor to interrupt their bodily integrity by cutting into them, injecting them, or procedurally “invading” their organs. In service of those unique roles, surely, we should want exceptionally high standards from our clinicians. Deep reflection and formation of conscience throughout clinicians’ careers regarding the morality of their actions is called for, to the benefit of all citizens.^12^
5. The ability to exercise conscience and resolve moral distress helps keep clinicians and leaders healthy and whole. That attention to worker health is a “good” in itself and is a value that many health systems ascribe to.^11,12,19,25^

So, what ought health leaders do? In ethics considerations, we focus our attention on respecting the autonomy of individuals to decide and influence the course of their lives, we support fairness and equity, and we optimize benefits and minimize harms. The complexity of healthcare does not allow resolution of all ethical dilemmas with reference to just these principles, however. Choices must be made in clinical and management settings, and in policy and regulatory decisions. We look to additional principles to assist, such as seeking the least intrusive means that achieves desired objectives. We are also guided by the principle that invoked measures should be in proportion to the degree of benefit and the risks of harm to individuals and to populations. These latter two considerations are especially important when addressing highly contested issues, such as assisted death.

When there is an available mechanism to adequately satisfy the competing core imperatives, there is no rational justification for offending either. The Province of Alberta, to use an example, has deliberately generated a readily accessible mechanism—the Medical Assistance in Dying Care Coordination Service—so that any person can receive information about, and facilitation for, any of the steps leading to potential provision of assisted death.^28,^^(note b)^ This includes access to information, eligibility assessments, transfers if required, provision of properly constituted chemicals, the practitioner to administer them, paperwork completion, and post-provision support. Since this rigorous service is widely available, there is a reduced duty on the part of any individual clinician to facilitate or provide assisted death.^25^ In other words, the “system” assures availability, so that individual clinicians are not compelled to participate. However, if a particularly isolated patient truly has no reasonable means to access this service—and this presumably would be a rare circumstance—there is a coincident duty on the part of the patient’s clinician, even if objecting to participation for reasons of conscience, to recognize this potential barrier and facilitate in some way a connection between the requesting patient and the Care Coordination Service.

To the degree that it is important enough to not offend either of the necessary but competing imperatives of assuring access while not diminishing conscience claims, the effort and cost to put such a mechanism in place is justified. This is a proportionate and least intrusive policy response.

An argument surfaces regarding the bounds of what can be regarded as a legitimate and a reasonable claim to conscience.^12,26,29^ I will not address possibilities for procedural mechanisms to adjudicate or appeal claims of conscience, although dedicated thinking about such mechanisms needs to occur and some have begun to address this.^29^ Rather, I will briefly list candidate substantive criteria that must be fulfilled for a conscientious objection to gain legitimacy, as proposed by various authors. These focus on the genuineness or sincerity of the claim, and the reasonableness of objecting:

a) the position should arise from deep consideration and conviction^12,26^;
b) it should be based on an account of the facts that are accurate^26,29^;
c) the result of provision would constitute a wrong that is very serious^26^ and would cause serious moral harm to the clinician^12^;
d) the moral position underpinning the conscientious objection is plausible^12^ such that others could accept it and act similarly,^29^ and those who oppose the position can still respect it as a carefully considered one^26^;
e) the reasons for objection cannot be discriminatory about the patient^29^; and
f) the specific intervention is not an essential role of the clinician’s specific practice.^12^

This list, I want to claim, comprises necessary considerations to satisfy genuineness and reasonableness conditions of a conscience claim.

Finally, and specifically to assisted death and the acceptability of conscience claims, actions have been proposed that should be invoked and some that should be avoided when a clinician objects for reasons of conscience. These include providing accurate information, not impeding access by another clinician, minimizing serious burdens to patients and colleagues, approaching the situation compassionately, and being open about one’s own limits without making the patient feel judged.^12,26,29,30^

## Conclusion

There is ongoing debate regarding the degree to which objections to provision of healthcare services, for reasons of conscience, are supportable. Some argue that conscience claims should have either no place or a subjugated place vis-a-vis patient access rights. However, we ought to be concerned that subjugation of conscience adherence in healthcare practice, when viewed outside of the platform of competing individual rights, will risk extinguishing the nuanced wisdom and early warning mechanism that the discernment and exercise of conscience affords. Healthcare is elevated, benefiting all members of society, when clinicians actively form and continually consider their consciences, and act in adherence to their moral commitments. Appropriate access to services in respect of patients’ autonomous choosing should also be assured. There are ways to accomplish both imperatives within assisted death programs, and current exemplar programs that do so are in place in at least some jurisdictions in Canada. Objective substantive criteria have been proposed to determine what might count as genuine, reasonable, and acceptable conscience claims. Work is required to develop procedurally sound mechanisms to adjudicate such claims.

## Notes

a. The author was a member of the committee that wrote the current Canadian Medical Association Code of Ethics and Professionalism.

b. The author served in the past as paid Medical Advisor and Secretariat Chair for the development and implementation of the MAiD mechanism in Alberta that was created in response to the federal legislation.
